# Irritability in pre-clinical Huntington's disease

**DOI:** 10.1016/j.neuropsychologia.2009.10.016

**Published:** 2010-01

**Authors:** Stefan Klöppel, Cynthia M. Stonnington, Predrag Petrovic, Dean Mobbs, Oliver Tüscher, David Craufurd, Sarah J. Tabrizi, Richard S.J. Frackowiak

**Affiliations:** a Department of Psychiatry and Psychotherapy, Section of Gerontopsychiatry, Section of Neuropsychiatry, Freiburg Brain Imaging, University Clinic Freiburg, Freiburg, Germany; b Wellcome Trust Centre for Neuroimaging, Institute of Neurology, UCL, London, UK; c Division of Adult Psychiatry, Mayo Clinic, Scottsdale, AZ, USA; d Department of Clinical Neuroscience, Karolinska Hospital, Stockholm, Sweden; e MRC Cognition and Brain Sciences Unit, Cambridge, UK; f Department of Neurology, Neurozentrum, Freiburg Brain Imaging, University Clinic Freiburg, Freiburg, Germany; g Academic Unit of Medical Genetics and Regional Genetic Service, St Mary's Hospital, Manchester, UK; h Department of Clinical Neurology, Institute of Neurology, UCL, London, UK; i Service de neurologie, Centre hospitalier universitaire vaudois et Université de Lausanne, Switzerland; j Laboratory of Neuroimaging, IRCCS Santa Lucia, Roma, Italy

**Keywords:** Huntington's disease, Irritability, fMRI, Amygdala, Orbitofrontal cortex

## Abstract

Irritability, together with depression and anxiety, form three salient clinical features of pre-symptomatic Huntington's disease (HD). To date, the understanding of irritability in HD suffers from a paucity of experimental data and is largely based on questionnaires or clinical anecdotes. Factor analysis suggests that irritability is related to impulsivity and aggression and is likely to engage the same neuronal circuits as these behaviours, including areas such as medial orbitofrontal cortex (OFC) and amygdala.

16 pre-symptomatic gene carriers (PSCs) and 15 of their companions were asked to indicate the larger of two squares consecutively shown on a screen while undergoing functional magnetic resonance imaging (fMRI). Despite correct identification of the larger square, participants were often told that they or their partner had given the wrong answer. Size differences were subtle to make negative feedback credible but detectable.

Although task performance, baseline irritability, and reported task-induced irritation were the same for both groups, fMRI revealed distinct neuronal processing in those who will later develop HD. In controls but not PSCs, task-induced irritation correlated positively with amygdala activation and negatively with OFC activation. Repetitive negative feedback induced greater amygdala activations in controls than PSCs. In addition, the inverse functional coupling between amygdala and OFC was significantly weaker in PSCs compared to controls.

Our results argue that normal emotion processing circuits are disrupted in PSCs via attenuated modulation of emotional status by external or internal indicators. At later stages, this dysfunction may increase the risk for developing recognised, HD-associated, psychiatric symptoms such as irritability.

## 1 Introduction

Huntington's disease (HD) is an inherited neurodegenerative disorder caused by an expanded number of triplet repeats of the nucleotide bases cytosine, adenine and guanine (CAG) in the gene encoding the protein huntingtin (HD Collaborative Research Group, 1993). Irritability, together with depression and anxiety, form a triad of core psychiatric features of pre-symptomatic HD. Irritation is defined as a temporary psychological state characterised by impatience, intolerance, and poorly controlled anger. It includes elements of anger, aggression and reduced impulse control and can occur independently of depression (Snaith, Constantopoulos, Jardine, & McGuffin, 1978).

To date, studies of irritability in patients with HD have relied on questionnaires. A recent study found increased levels of irritability in around 20% of pre-symptomatic gene carriers (PSCs) with less than 10 years to estimated diagnostic onset who were unaware of their gene status (Julien et al., 2007). Irritability causes great distress to those close to HD patients and often determines if somebody can be managed in the community or needs to be admitted to a nursing home (Folstein, Chase, Wahl, McDonnell, & Folstein, 1987; Hamilton et al., 2003; Wheelock et al., 2003).

Factor analysis suggests that irritability in HD is related to impulsivity and aggression (Craufurd, Thompson, & Snowden, 2001). The amygdala and medial orbitofrontal cortex (OFC) are key circuits involved in impulsive aggression (Blair, 2007; Davidson, Putnam, & Larson, 2000; Siever, 2008). This notion was suggested in studies focusing on structural changes (Anderson, Bechara, Damasio, Tranel, & Damasio, 1999; Tebartz van Elst, Woermann, Lemieux, Thompson, & Trimble, 2000) and later confirmed by neuropsychological and functional imaging studies (Best, Williams, & Coccaro, 2002; Coccaro, McCloskey, Fitzgerald, & Phan, 2007; Dougherty et al., 2004). A growing body of evidence suggests an inverse correlation between these two areas (Coccaro et al., 2007; Dougherty et al., 2004; Urry et al., 2006) with the medial OFC exerting an inhibitory influence on the amygdala, most likely through direct anatomical connections (Rempel-Clower, 2007).

Neuropathological as well as imaging studies indicate an involvement in HD of structures implicated in the regulation of aggression, including the amygdala (Douaud et al., 2006; Mann, Oliver, & Snowden, 1993; Pavese et al., 2003; Rosas et al., 2003) and prefrontal cortex (Pavese et al., 2003; Rosas et al., 2002) but there is little indication of a specific involvement of the OFC, at least in earlier stages of the disease. Although the neuronal mechanism is not fully understood, a role for the serotonergic system in impulsive aggression has been suggested (Coccaro & Kavoussi, 1997). The very limited available data indicate serotonin reuptake inhibitors could be useful for the treatment of irritability in HD (De Marchi, Daniele, & Ragone, 2001; Ranen, Lipsey, Treisman, & Ross, 1996) as an alternative to atypical neuroleptics (Paleacu, Anca, & Giladi, 2002; Squitieri et al., 2001).

Our study therefore aimed to examine the emotional circuitry associated with induced irritation with a focus on the amygdala. Firstly, we intended to develop a task capable of reliably causing irritation in participants and to study the neuronal correlates of such a task with functional MRI (fMRI). Although PSC do not show gross impairments we sought to design a task that can easily be performed in an fMRI environment and does not require substantial dexterity or rely heavily on working memory performance. Unmatched task performance could have made the interpretation of differences in neuronal processing difficult. A number of studies in PSC have shown altered neuronal processing in the absence of differences in task performance (Kloppel et al., 2009; Reading et al., 2004; Wolf, Vasic, Schonfeldt-Lecuona, Landwehrmeyer, & Ecker, 2007). In line with these studies, we expected changes in neuronal processing associated with an experimental task that induces irritation, even in those PSCs without clinically increased irritability. As outlined above, we assumed irritability in HD to be related to impulsive aggression and influenced by social interaction. Based on previous work in subjects with intermittent explosive disorder (Coccaro et al., 2007), a disease characterized by impulsive aggression, we hypothesized that our experimental task would elicit increased amygdala activations in PSCs and a disruption of amygdala-OFC coupling. Our clinical experience, and that reported by others (Snowden et al., 2003), indicates that individuals with the HD gene mutation get particularly irritated with close companions. We therefore expected greater activations of the amygdala when PSCs lost a round due to a mistake by their companion compared to trials lost by mistakes from a computer.

## 2 Material and methods

### 2.1 Participants

16 PSCs and their close companions (14 partners, one close friend and one same-generation relative) were included. PSCs were aware of their gene status. As mentioned, we decided to include companions who are indirectly affected by the disease who therefore do not represent the general population. We reasoned that the inclusion of companions would illicit the strongest levels of irritability. An assumption which was based on our clinical experience as outlined in the introduction.

PSCs comprised a wide range of estimated years to clinical diagnosis, based on age and the number of CAG repeats (Langbehn, Brinkman, Falush, Paulsen, & Hayden, 2004). With the exception of the one relative, companions did not undergo genetic testing but none had a family history of HD or showed clinical signs. Other than one control subject who had isolated seizures under the age of 12, there were no medical co-morbidities. Two PSCs had previously taken antidepressant medication for more than a year (one took citalopram 30 mg; the other could not recall the name of the substance or dosage), but none were actively using antidepressants at study. One PSC was taking 10 g creatine daily. No one else had a history of neurological or psychiatric disorder and none had used centrally acting medication. A neurologist experienced in HD examined all PSCs. One control failed to undergo scanning and so was excluded from all analyses. The remaining subjects were matched for age, gender, National Adult Reading Test (NART) score (Nelson & Willison, 1991) and years in education (Table 1). The local Ethics Committee approved the study and all participants gave written informed consent according to the Declaration of Helsinki.

### 2.2 Experimental procedure

**Fig. 1:**
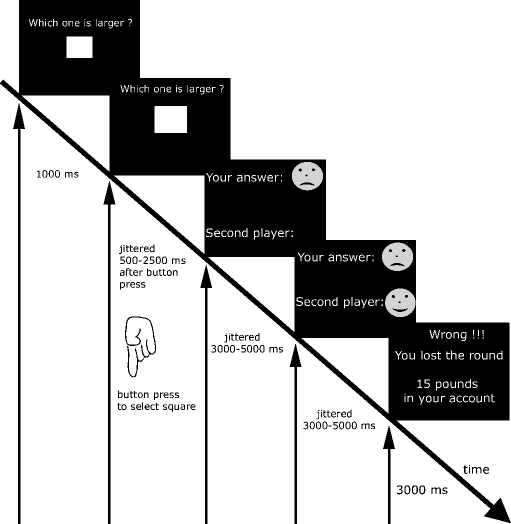
Overview of task. Subjects had to identify the larger of two squares shown sequentially. This was followed by feedback on the correctness of the answer of the first and second player. A separate screen indicated if the round was won (when both players answered correctly) or lost. Timing intervals between screens were randomised (jittered) where indicated.

The task was designed to study two factors. Firstly, we sought to study neuronal processing with false allegations of one's own performance errors. We expected the induced irritation to build up with repetition of these false allegations. The second factor under study was the identity of the second player. Based on clinical experience, we expected a stronger emotional response when a round was lost by a companion's mistake than that made by a non-human (i.e., a computer). Both factors were integrated into the same task in which two squares were shown consecutively to participants who were asked to identify the larger (see Fig. 1 for details). A total of 50 trials per run were performed.

In a pilot phase with healthy participants performing different versions of the task we identified a difficulty level that resulted in correct answers on most trials. We found that it is much harder to correctly compare the size of two objects when the first object shown is larger. We therefore ended up with three different squares for the study. If a smaller square was shown first, it measured 2.8 cm edge length and was followed by a larger square of 2.9 cm. A square of 3.1 cm edge length was followed by the square of 2.8 cm edge length. Slight differences of the combined distances between screen and mirror and mirror and eye were tolerated to ensure comfortable positioning of subjects and an unblocked view of the screen. Based on interviews with participants of the pilot study we determined that the task remained credible when 28% of correct answers were followed by error as feedback. This number was found through trial and error in runs limited to exactly 50 trials. Written instructions stated that the study ‘examines how the brain responds when doing tasks and getting feedback’. We deliberately avoided any mention of irritability in the study description, as that might have altered emotional responses and neural processing.

#### 2.2.1 Feedback on own performance

**Fig. 2:**
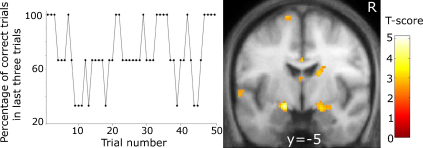
Left: The graph displays the changing percentage of positive feedback in the last three trials for each run given that a subject's true answer was always correct. Right: Bilaterally the amygdala showed significantly greater activations in controls compared to PSCs when subjects were repeatedly given feedback that they had chosen the wrong square.

We hypothesised that subjects would experience irritation after being informed they had made a mistake in a task they were sure they had performed correctly. Furthermore, we expected the induced irritation to build up with repetition of false allegations of erroneous performance. We therefore varied the percentage of conflicting feedback responses over the course of the 50 trials as shown in Fig. 2 (left panel). The variation was constrained by avoiding presentations with more than two consecutive trials comprising incorrect negative feedback.

#### 2.2.2 Identity of the second player

Subjects were led to believe that their computer was linked to their partner's computer who was playing simultaneously in another room and that both players had to answer correctly to win a round. Three pounds were added to a player's account for every round won and deducted again when a round was lost. For comparison and to allow full balancing of the design, an additional ‘second player’ in the form of a computer was introduced which would try to simulate the behaviour of a human and could therefore make mistakes. Playing with a computer was found less emotionally involving in our pilot study which mirrors results of others (Rilling, Sanfey, Aronson, Nystrom, & Cohen, 2004).

After a brief training period with correct feedback, each subject performed four runs of 50 trials. Two runs (one with a partner and the other with a computer) were performed in the MRI scanner. The other two were performed in a testing room using a standard PC. Participants swapped rooms after two runs. The order of runs and whether PSCs or controls started in the MRI scanner was randomised over the study.

#### 2.2.3 Ratings and questionnaires

After training, subjects were asked to indicate on a visual analogue scale (VAS), ranging from −100 (indicating worst performance) to +100, how well they expected they and their partner would do. Using a similar VAS after every two or three rounds, subjects were asked how confident they were with their performance in the task. After the experiment, subjects completed the Snaith irritability self-assessment scale (Snaith et al., 1978), Beck Depression Inventory (BDI; Beck, Ward, Mendelson, Mock, & Erbaugh, 1961), Barratt's Impulsivity Scale (BIS-11; Barratt & Patton, 1983) and Spielberger State-Trait Anxiety Inventory (STAI; Spielberger, 1988). As PSC might be reluctant to admit their true level of irritability we adopted the approach of Chatterjee, Anderson, Moskowitz, Hauser, and Marder (2005) by evaluating discrepancies between PSC's self rating and that of their companions using the Johns Hopkins irritability questionnaire. Results between the two groups on the 14 item questionnaire were compared using two-tailed paired *t*-tests. We refer to this questionnaire as the Johns Hopkins irritability questionnaire. All items are listen in the study by Chatterjee et al. (2005). In addition, they completed a questionnaire of how they felt when receiving feedback. We assumed that each subject would have a different understanding of ‘irritation’. We therefore asked them to rate three negative emotions (irritation, anger, tension) ranging from zero (emotion not felt) to three (emotion strongly felt). Similarly, subjects were asked to report, using the same scale, their positive emotions (happy and relieved) on receiving positive feedback. We tested for a positive correlation of negative emotions (composite score of irritability, anger, and tension) induced by the task, with Snaith's score and the irritability subscore (summation of inward and outward irritability sub-scores) to identify a relation of the task to our concept of irritability. Similarly, since irritability is closely connected to problems with impulse control (Craufurd et al., 2001) scoring on the BIS-11 and task-induced negative emotions were also expected to be positively correlated. Significance was tested with non-parametric tests when significant Kolmogorov–Smirnov tests indicated a violation of assumptions for parametric testing.

Before detailed debriefing, all participants took part in a semi-structured interview, one purpose of which was to verify that they had believed in the integrity of the task, the link with the other player and the correctness of feedback.

### 2.3 MRI-scanning

We used an echo planar imaging sequence optimised for sensitivity in amygdala and OFC (Deichmann, Gottfried, Hutton, & Turner, 2003) scanned on a 1.5 Tesla MRI system (Sonata; Siemens, Erlangen, Germany) at a single centre. Scanning parameters included: repetition time 3600 ms; echo time 50 ms; field of view 192 mm; distance factor 50%; flip angle 90°. We used 40 slices with a thickness of 3 mm angled at 30°. We sought to measure the individual distortions in OFC and amygdala due to the close proximity of air-filled cavities by acquiring field maps (Hutton et al., 2002). Total scanning time for the fMRI task was around 30 min per subject depending on speed of responses. An additional T1-weighted sequence (Deichmann, Schwarzbauer, & Turner, 2004) was acquired to exclude structural abnormalities and to evaluate structural differences using voxel-based morphometry (Ashburner & Friston, 2000) (see supplementary material).

### 2.4 Image data analysis

Pre-processing and statistical analysis of fMRI data were carried out using SPM5 software (www.fil.ion.ucl.ac.uk/spm/). Before smoothing with an 8 mm Gaussian kernel, volumes were realigned and spatially normalised to a standard echo planar imaging template in Montreal Neurological Institute (MNI) space. Field maps were included in the realignment step. A first-level analysis based on the general linear model (Friston, Frith, Turner, & Frackowiak, 1995) was performed for each subject. A 128 s high-pass filter was applied. Task-related changes in fMRI signal were estimated at each voxel by modelling the onsets and length of each event type as a separate regressor convolved with a haemodynamic response function. For our primary analysis, we used four regressors to model the onset of positive and negative feedback, for the first and second player respectively. They were modelled as mini-blocks with a length of 3–5 s, depending on the onset of the next screen (see Fig. 1). Since we were interested in the effect of repeated negative feedback, we entered a separate parametric modulator to both regressors of negative feedback, coding how often subjects had received negative feedback in the last three trials. As illustrated in Fig. 2 the modulator thus contains values of either 100 when positive feedback was received for all three previous trials 66 or 33 when only one trials of the last three returned positive feedback. We did not aim to evenly distribute these percentages as including too many trials with false negative feedback would have made the task less believable. All four feedback conditions had separate parametric modulators assigned to model linear effects of time (e.g., due to fatigue). Error rates were low and for a subject not making a real mistake 36 correct trials were contrasted with 14 trials in which false allegations of erroneous performance was reported. An illustration of the design matrix is provided as supplementary material. As shown, the parametric modulator coding past negative feedback is assigned to the negative feedback condition only. Additional regressors were included to model button presses, the different screens as well as six regressors obtained at the realignment step to account for movements (translations in three planes and rotations along three axes). Trials when a player made a real mistake were excluded from further analysis. We reasoned that cognitive processing of such trials would differ from those where negative feedback followed correct performance. In the case of true mistakes, participants might doubt their answers while negative feedback in the second case is likely to be more unexpected. The resulting set of voxel values for each contrast entered a second level analysis.

### 2.5 Group-level random effects analysis

#### 2.5.1 Feedback on own performance

Parameter images of the differential effect of negative compared to positive feedback entered a 2-sample *t*-test with 28 degrees of freedom resulting from 30 participants in two groups. A similar design was employed to study parameter estimates from the parametric modulator coding the percentage of negative feedback in the last three trials (Fig. 2). As explained above, this analysis tests the hypothesis that emotions build up when subjects repeatedly receive negative feedback, despite correct performance.

#### 2.5.2 Correlational analysis

We expected that subjects with a higher level of task-induced irritability reported in questionnaires would show greater activations in the amygdala when negative feedback was compared to positive feedback. We also correlated the parameter images from the comparison of negative and positive feedback and those from the parametric modulation of the percentage of negative feedback in the last three trials with the estimated years to onset and included age as a separate regressor. As before, we expected increased activations of the amygdala with approaching diagnostic disease onset and a higher frequency of erroneous negative feedback.

#### 2.5.3 Regions of interest

The primary focus of the feedback and correlational analyses described above was the amygdala, defined using the “anatomy toolbox”, based on post-mortem tissue analysis (Eickhoff et al., 2005). We created a region of interest including all voxels with at least a 50% probability of belonging to the amygdala. The second focus was the medial OFC for which no such template is currently available. We therefore created a sphere with a radius of one cm around the most activated voxel in the medial OFC from the control group of a recent study on impulsive aggression (at *x*, *y*, *z* =  ± 6, 52, −20 in MNI space) (Coccaro et al., 2007). Correction for multiple comparisons within each of the regions was performed using the false discovery rate (FDR) as implemented in SPM5 at a critical *p*-value of 0.05 at the voxel level. Outside our regions of interest voxels are reported if they survived FDR correction performed across the whole brain.

#### 2.5.4 Time series analysis

As in a related study (Coccaro et al., 2007), we performed an additional analysis to test for a difference in the strength of the negative correlation between amygdala and medial OFC that depends on gene status. Based solely on the fMRI time series (and not on the parameter images used in other analyses), this analysis tests whether activity in a brain region (i.e., the amygdala) correlates differentially with other brain regions depending on gene status. This analysis is complementary to the group-level analysis described above as it takes the fMRI time series from the whole experiment and not just data from one specific condition. The time-courses in the left and right amygdala were extracted from the centre of the probabilistic atlas of the amygdala (Eickhoff et al., 2005) (centro-lateral segment) (*x*, *y*, *z* = −25, −9, −18 and 29, −8, −19 in MNI space). The time series was used as a single regressor in subsequent analysis. The resulting parametric images entered a second level 2-sample *t*-test analysis as described above.

#### 2.5.5 Identity of the second player

We performed an exploratory three-way ANOVA with the factor GROUP (two levels: PSC and controls) and two repeated-measures factors, PLAYER (two levels: partner and computer) and CONDITION (four levels: positive/negative feedback on each of a pair of players). Of primary interest to our research question was the subsequent *F*-test that identified regions showing an interaction of GROUP by PLAYER. Based on previous studies (Gallagher, Jack, Roepstorff, & Frith, 2002; Rilling et al., 2004) on interaction of humans with computers we expected the dorso-medial prefrontal cortex (dmPFC) to show increased activity if the partner was human. FDR correction was performed in the same regions of interest as above. This analysis had a strong exploratory component and we thus report all regions within the frontal lobe significant at *p* < 0.001 without correction for multiple comparisons for the interaction of GROUP by PLAYER.

## 3 Results

### 3.1 Behavioural data and ratings

All participants, except one control and three PSCs, reported a negative emotion with negative feedback. No significant differences between the groups were found in any of the questionnaires including those testing irritability and impulsivity (Table 2). A correlation matrix of the questionnaires is provided as a supplement. Similarly, no significant differences between PSC irritability self-ratings and those of their companions were found using the Johns Hopkins irritability questionnaire (PSC on themselves (mean ± SD): 11.6 ± 6.3; companions on PSC: 10.5 ± 6.1; *p* > 0.5, paired *t*-test; data available from 15 pairs only). There were no significant differences between PSCs close to and far from estimated clinical onset except that those far from onset had a lower number of CAG repeats (*p* = 0.02). Four PSCs and three controls had mild depressive symptoms indicated by a BDI over 10. The rating on Snaith's irritability questionnaire was within the normal range (Snaith et al., 1978) in all but one PSC. Task-induced negative emotions (composite score of irritability, anger and tension) showed a significant positive correlations with both Snaith's score (*r*_Spearman_ = 0.31; *p* = 0.048) and BIS-11 impulsivity score (*r*_Spearman_ = 0.46; *p* = 0.005). Similarly, a trend for a positive correlation was found with the Snaith's irritability subscore (*r*_Spearman_ = 0.28; *p* = 0.064). Both groups performed equally well and were equally confident about their decisions (Table 2). There was a tendency that PSCs expected to perform worse than controls (*p* = 0.1). No correlations of scores in questionnaires with the estimated years to clinical onset were found. Interviews and debriefing confirmed that subjects believed in the correctness of feedback and the link between cooperating players.

### 3.2 Image data analysis

No significant activations in amygdala or OFC were observed when negative and positive feedback was compared for players in the scanner either separately for each group or for both groups combined. There was also no significant effect when testing for an interaction with group status inside one of the regions of interest or after correction across the whole brain. In a subsequent analysis we tested for increased neuronal responses in the presence of repeated negative feedback, despite correct performance. In controls, greater activation was found in the left amygdala (*T* = 3.79; *p* = 0.05 at *x*, *y*, *z* = −18, −6, −10) the higher the proportion of negative feedback. Neither the effect in the right amygdala of controls (*T* = 3.2 at *x*, *y*, *z* = 30, −2, −12) nor bilaterally in PSCs (*T* < 2.8) survived correction for multiple comparisons. A significant interaction with gene status was found in left amygdala and a strong trend in right amygdala resulted from PSCs failing to show the expected positive correlation with negative feedback (*T* = 4.02; *p* = 0.005; at *x*, *y*, *z* = −18, −6, −12 and *T* = 3.37; *p* = 0.06 at *x*, *y*, *z* = 18, −8, −14; Fig. 2). No differences between PSCs close to and far from clinical onset were found. There were also no differences between groups in the OFC region of interest. No further significant voxels were found when the search volume included the whole brain.

#### 3.2.1 Time series analysis

**Fig. 3:**
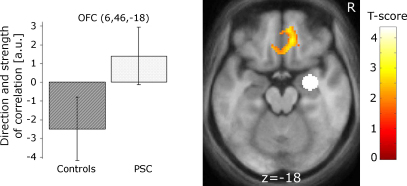
Areas showing a stronger negative correlation between right amygdala activity (white circle) in the control group compared to PSCs. The seed region in the amygdala is enlarged for visualisation purposes. The plots on the left display the strength and direction of coupling. Bars report the strength of correlation in arbitrary units (a.u.) with 90% confidence intervals. Imaging results are overlaid on the mean brain from all subjects in MNI space at a threshold, for visualisation purposes only, of *p* < 0.01 (uncorrected).

The significant interaction in the time series analysis resulted from greater negative coupling between activations in right amygdala and medial OFC in controls than in PSCs (*T* = 2.81; *p* = 0.05 at *x*, *y*, *z* = 6, 46, −18) (Fig. 3). The figure also illustrates group specific main effects, which did not survive at a corrected level. Activations did not differ significantly between PSC groups differing in proximity to clinical onset.

#### 3.2.2 Correlational analysis

**Fig. 4:**
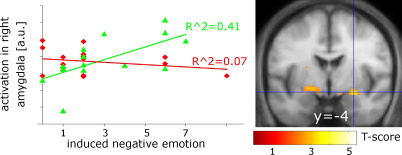
Areas showing an interaction between groups in the subject-specific rating of task-induced negative emotions. Whereas controls show increased activations in amygdala with increasing levels of reported negative emotions, such correlations are absent in the PSC group. Graphs show the correlation of rating with activation at the voxel marked by cross hairs in controls (green triangle) and PSC (red diamonds). Results are displayed at a threshold of *p* < 0.01 (uncorrected). a.u.: arbitrary units. (For interpretation of the references to color in this figure legend, the reader is referred to the web version of the article.)

Task-specific negative emotional ratings from controls correlated positively with the fMRI signal for negative vs. positive feedback bilaterally in the amygdala (*T* = 3.11; *p* = 0.05 at *x*, *y*, *z* = −20, −8, −10 and *T* = 3.77; *p* = 0.036 at *x*, *y*, *z* = 26, −4, −12) and there was also a significant group interaction in the right amygdala (*T* = 3.61; *p* = 0.018 at *x*, *y*, *z* = 16, −4, −16) (Fig. 4). Thus, higher levels of reported irritation resulted in stronger activations of the amygdala in controls compared to PSCs for which correlations were virtually absent (*T* < 1.5). Controls with lower levels of reported task-induced irritation showed higher neuronal activations in the right OFC (*T* = 4.64, *p* = 0.003 at *x*, *y*, *z* = 12, 52, −12) and a similar trend on the left (*T* = 2.6 at *x*, *y*, *z* = −10, 48, −12). No significant correlations with the reported ratings and activations in OFC were found for PSC and there was no significant interaction. In addition, we did not find significant correlations with the estimated years to diagnostic onset.

### 3.3 Effect of second player

**Fig. 5:**
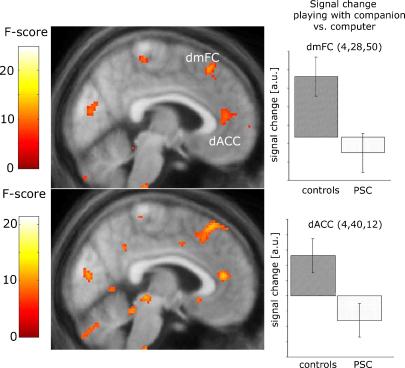
Effect of identity of the second player. The top left panel depicts areas where controls show increased activations when the second player is a human partner compared to a computer. The lower left panel indicates that this effect is weaker in PSCs, resulting in a positive interaction in both areas. The two right panels depict the signal change in both areas when partner and computer conditions are compared (co-ordinates are in MNI space). In both areas, PSCs have reduced activations when the second player is a companion. Error bars indicate 90% confidence intervals. Results are displayed at a level of *p* < 0.01 (uncorrected) for visualisation purposes. dACC: dorsal anterior cingulate cortex; dmPFC: dorso-medial prefrontal cortex; a.u.: arbitrary units.

In controls but not PSCs, two areas in the frontal lobe, the dorsal anterior cingulate cortex and dmPFC, showed differential activity depending on whether the second player was a partner or a computer (*F* = 9.32; *p*_(uncorrected)_ = 0.003 at *x*, *y*, *z* = 4, 42, 10) and *F* = 11.80; *p*_(uncorrected)_ = 0.001; at *x*, *y*, *z* = 4, 28, 50) (Fig. 5). Neither significant effect was found in amygdala or OFC region of interest, nor was there a significant interaction in these regions. Very similar areas were found when testing for an interaction of GROUP with PLAYER (*F* = 12.01; *p*_(uncorrected)_ = 0.001 at *x*, *y*, *z* = 4, 40, 12) and (*F* = 13.4; *p*_(uncorrected)_ < 0.001 at *x*, *y*, *z* = 8, 34, 56) (Fig. 5). An additional peak was found in the right dorso-lateral prefrontal cortex (*F* = 13.97; *p*_(uncorrected)_ < 0.001 at *x*, *y*, *z* = 28, 26, 40). No significant interactions of GROUP with PLAYER or GROUP with PLAYER and CONDITION were found in amygdala or OFC.

## 4 Discussion

### 4.1 Behavioural data

We found equal scorings on ratings or questionnaires by PSCs and controls. Both groups scored within the normal range on the Snaith irritability questionnaire. A recent study suggests that clinically overt irritability is found in around 20% of PSCs who are less than 10 years to estimated clinical onset, but is rare before that (Julien et al., 2007). Our sample contained only three subjects with less than 10 years to onset and was not pre-selected for indications of increased irritability. Furthermore, companions may have been more “stressed” than unrelated controls. No differences between PSC self rating of irritability and those of their companions were found which mirrors findings from the study of Chatterjee et al. (2005). The BDI identified subjects with mild or moderate depressive symptoms (BDI > 10) in both groups, potentially reflecting the emotional burden of pre-symptomatic HD on close companions as well as PSCs.

Equal levels of self-reported task-induced irritation associated with differential group specific neural responses suggest that either compensatory mechanisms are in place or that the irritability questionnaires lacks sensitivity to detect subtle differences between groups. Their improvement is an important part of ongoing research.

### 4.2 Feedback on own performance

Our fMRI analysis leads us to reject our first hypothesis regarding task-induced amygdala activations. PSC did not show greater activations in the amygdala than controls when negative feedback was compared to positive feedback. We found no indication for differential activations in the OFC/amygdala axis with the identity of the second player. We can, however confirm the hypothesis of a reduced coupling between amygdala and medial OFC in the PSC group.

All three imaging analyses indicate that neuronal responses in PSCs were modulated less by external (i.e., the proportion of erroneous responses) and internal factors (i.e., the level of task-induced irritation) than in controls. Increasing false negative feedback (indicated either by subject-specific ratings or a higher frequency of negative feedback) resulted in increased activations in overlapping areas of the amygdala in controls, whereas this effect was reduced or absent in PSCs. We argue that the inappropriate responses of the amygdala/medial OFC axis make PSCs prone to the development of psychiatric symptoms such as irritation.

### 4.3 Time series analysis

A disruption of the amygdala/medial OFC axis has been found in recent work on impulsive aggression (Coccaro et al., 2007) and also in subjects suffering from depression with anger attacks (Dougherty et al., 2004). Early involvement of the amygdala in HD-related pathology (Douaud et al., 2006; Rosas et al., 2003) could be the basis of the reduced coupling and responsiveness to reported levels of experienced negative emotions. The similarity of findings to aggressive disorders lends support to the presence of an aggressive element in HD, which has also been suggested by neuropsychological factor analysis (Craufurd et al., 2001). While the aforementioned studies included patients with psychiatric symptoms (Coccaro et al., 2007; Dougherty et al., 2004) our PSCs showed normal levels of irritability as measured by the Snaith scale (Snaith et al., 1978).

Reduced functional coupling could limit the ability of PSCs to relate to the strength and value of an emotion (Anderson et al., 2003). At least for the emotion of fear, it has previously been reported that under conditions of diminished conscious emotional awareness there is a decrease of connectivity between amygdala and cortical association areas (Williams et al., 2006). PSCs could be failing to recognise their own emotional state or might be denying it, a notion that is supported by a study comparing symptom ratings between HD patients and collaterals (Chatterjee et al., 2005; Hoth et al., 2007).

### 4.4 Effect of second player

Based on clinical experience, we hypothesised that irritability in PSCs is particularly pronounced in their interactions with close companions. No clear indication for this hypothesis was found by interview. Both groups consistently reported that playing with their partner was more emotionally involving but that this was the case for both positive and negative feedback. The interactive element of our task was far less pronounced than in other studies (Rilling et al., 2004); although the outcome of a given round depended on both players, the response of one player did not depend on that of the other. This is likely to have reduced the effect of the second player's identity.

The dorsal anterior cingulate cortex and dmPFC have been implicated in social interaction (Amodio & Frith, 2006). These regions were indeed activated by controls when playing with a partner rather than a computer, whereas this effect was absent in PSCs. This finding could represent a reduction in the ability to take the perspective of others, so that the identity of a second player mattered more to controls than to PSCs. A complimentary view is that dmPFC areas represent others’ views of the self (Amodio & Frith, 2006). Impairment in assessing what other people think about oneself (e.g., as when the self gets false negative feedback) could make one more prone to aggressive and disinhibited behaviour. However, these findings should be interpreted cautiously since they stem from an exploratory analysis and the interview results provide little to differentiate between the two possible explanations.

Although instructions stated that both players needed to give correct answers in order to win a round and the money, three PSCs and controls perceived the task as a “friendly competition” rather than a collaborative effort. Such heterogeneity is likely to reduce sensitivity for detection of differences between the two groups, especially in amygdala. We confirmed our pilot study as subjects experienced irritation with the task and believed in the ‘correctness’ of feedback and the ‘link’ between computers, but did not pick up this ambiguity.

### 4.5 Limitations

A number of aspects of the current study should be noted. Despite a trend for PSCs expecting to perform worse prior to engaging in the task, both groups actually performed equally well, with similar levels of induced positive and negative emotions and confidence in their decisions. A task with low levels of cognitive demand helped to ensure that subtle cognitive impairments in PSCs were not a source of between-group differences, making the observed differences in neuronal activations more likely to reflect the disease process and compensation for it. Notably, PSC in our study were aware of their genetic status which is likely to have influenced them at the behavioural and neuronal levels. Given the widespread availability of genetic testing, this situation is likely to represent the standard situation in a clinical setting when dealing with irritability and the study aimed to understand processing in these circumstances. More to the point, most studies with at-risk subjects ignorant of their genetic status were performed after subjects had decided to undergo genetic testing. The uncertainty and significance of the testing procedure, that includes mandatory counselling, could themselves modify clinical presentations of HD-related irritability.

As pointed out in the introduction, there is some evidence for structural changes affecting the amygdala. Although the analysis of the structural data acquired for the current study (see supplementary material) failed to identify changes in the structures identified with functional imaging, it is possible that structural changes underlie some of the functional differences found via remote effects.

The relative absence of studies on irritability in either healthy or diseased populations made it necessary to base this study on hypothetical assumptions. While the relationship of irritability to impulsive aggression is supported by a factor analysis (Craufurd et al., 2001), other clinical presentations of HD, including poor sleep and cognitive dysfunction, may play an important role and were not evaluated. As mentioned in the Methods section, each subject may have a different concept of irritability. Subjects were therefore asked to rate the intensity of three negative emotions (irritability, anger and tension). The significant positive correlation found between these emotions and the impulsivity and irritability questionnaires illustrates that the task is at least related to the presented concept of irritability. Given that irritability co-occurs with depression and anxiety and HD and that the results from the respective questionnaires were correlated with each other (see correlation matrix in the supplement) we cannot claim to be looking at irritability specifically and exclusively. Future work could focus on subjects pre-selected for increased levels of irritability to see if results are similar in quality and if the effect of contributing factors such as depression poor sleep and cognitive dysfunction can be isolated. The recording of additional physiological data, e.g., heart rate or skin conductance could also prove useful to find effects related to irritability.

All questionnaires were completed after the experiment to minimize the effect of reflecting on emotions from influencing behaviour and cognitive processing as the experiment progressed. This retrospective nature could have inflated correlations between questionnaires that were or were not related to the task (e.g., Snaith's questionnaire). Finally, a number of the imaging results we report are not significant after correction for multiple comparisons. We report them descriptively to illustrate that findings in amygdala and OFC are very probably bilateral and to generate hypothesis of the processing of the second player's identity.

## 5 Conclusion

In conclusion, our results indicate a poor dependency of neuronal responses on reported emotions or the quality of recent feedback in PSC. The disruption of emotion processing circuits may well predispose to the development of recognised psychiatric states, such as irritability. There is evidence that the processing of facial expressions of negative emotions is affected already in PSC (Gray, Young, Barker, Curtis, & Gibson, 1997; Johnson et al., 2007) and that dysphoria inducing pictures elicit differential brain activations in early affected HD patients compared to controls (Paradiso et al., 2008). Future research could clarify if these impairments of emotion processing predispose one to the clinical manifestation of irritability.

## Competing interests

None

## Funding

This work was supported by the Wellcome Trust (grant 075696 2/04/2 to R.S.J.F., and S.J.T.) and the Bundesministerium für Forschung und Gesundheit (BMBF grant 01GW0730 to O.T.). While writing the manuscript S.K. was funded by the Medizinische Fakultät of the University of Freiburg. P.P. is supported by a grant from Vetenskaps rådet and Hjärnfonden, Sweden.

## Figures and Tables

**Table 1:** Demographic and basic cognitive details of participants. Results are reported with mean and standard deviation.

	PSC	Controls	*p*-Values
*N*	16	15	NA
Gender (f/m)^b^	8/8	8/7	0.8
Age	39.3 ± 7.9	40.4 ± 90.4	0.73
CAG	42.14 ± 2.2^a^	NA	NA
Years to estimated onset	16.8 ± 8.8	NA	NA
Years in education	14.9 ± 2.9	15.6 ± 3.1	0.57
UHDRS-motor	2.3 ± 1.5	NA	NA
NART	104.2 ± 10.2	108.9 ± 9.0	0.18

a Exact CAG length missing from two subjects measured with different equipment.

b Chi-Square test for gender.

**Table 2:** Details of task performance and questionnaires completed after the experiment. Results are reported with mean and standard deviation unless stated otherwise.

	PSC	Controls	*p*-Values
BIS total	62.0 ± 11.8	63.3 ± 8.9	0.71
STAI (state)	34.0 ± 12.6	31.7 ± 8.9	0.57
STAI (trait)	34.2 ± 10.4	38.0 ± 10.8	0.32
BDI	5.7 ± 5.9	6.9 ± 6.1	0.59
Snaith total	13.2 ± 6.4	13.3 ± 5.5	0.95
Snaith irritability subscore	5.6 ± 3.3	5.5 ± 2.9	0.96
Negative emotion with loosing (max = 9)^a^	2.0 (0–9)	2.0 (0–7)	0.22
Positive emotion with winning (max = 6)	3.5 ± 1.5	3.7 ± 1.5	0.55
Correct responses [%]	93.8 ± 4.9	93.8 ± 4.9	0.99
Expected performance of self (min = −100, max = +100)	17.4 ± 30.0	36.7 ± 33.1	0.1
Expected performance of 2nd player (min = −100, max = +100)	57.6 ± 25.0	49.0 ± 25.5	0.35
Confidence in answer (min = −100, max = +100)	29.4 ± 25.8	38.7 ± 32.6	0.33

a Reported with median and range; Mann–Whitney test for between-group comparison.
