# Supplementary material for: Irritability in pre-clinical Huntington's disease
Source: Neuropsychologia. 2010 Jan;48(2):549–57. doi: 10.1016/j.neuropsychologia.2009.10.016 (PMC2809920; doi:10.1016/j.neuropsychologia.2009.10.016)
Supplement: Supplementary file 1 [file mmc1.doc]

Supplement 1: First level-design matrix


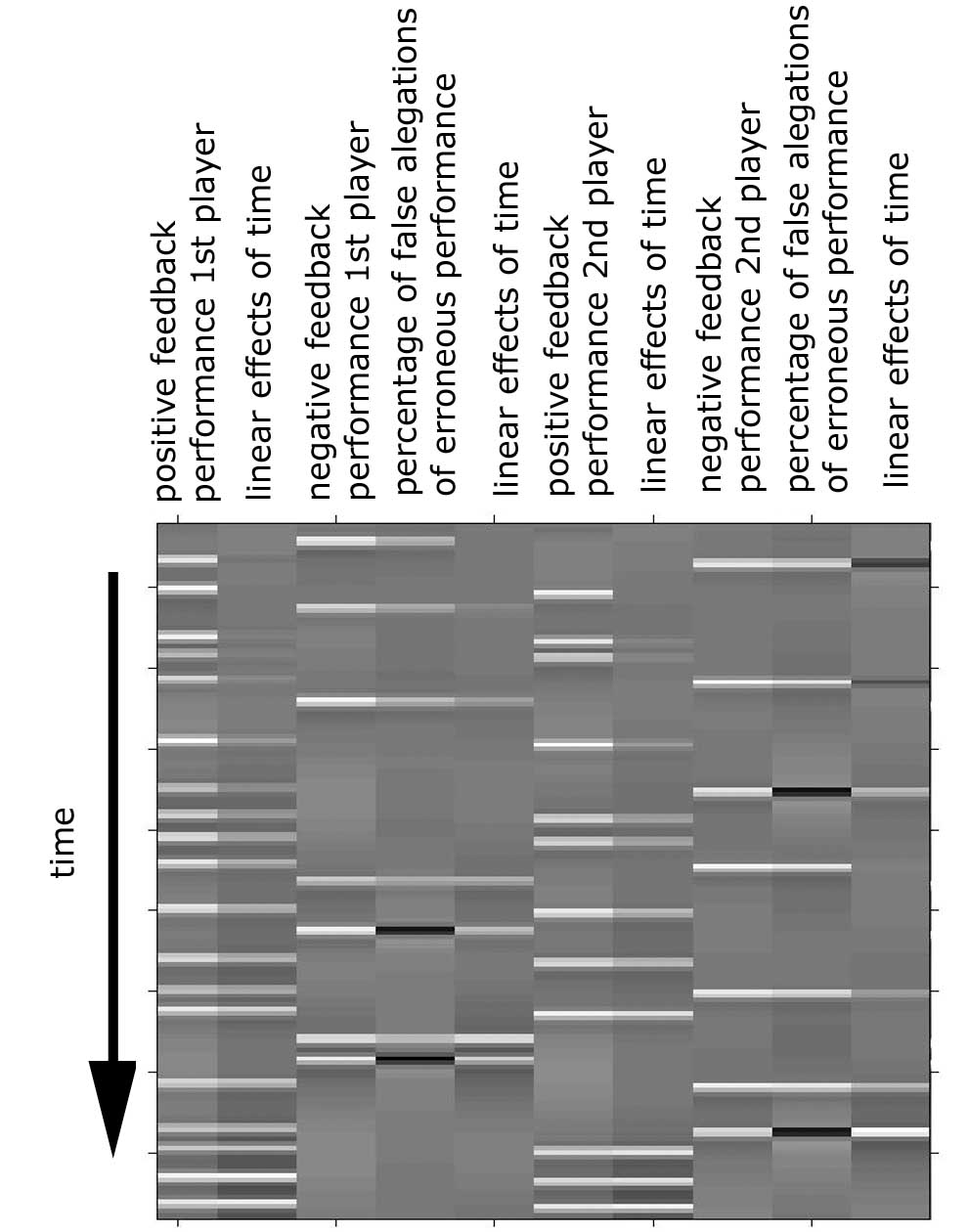


Supplementary figure 1: The figure displays a section of the design matrix with the four main regressors coding the different feedback conditions (positive and negative feedback for the 1st and 2nd player). The 1^st^ player is the one who actually performs the task. This player is led to believe that computers are linked and that the performance of the 2^nd^ player is the actually performance of either the companion or a computer.

Based on our initial hypothesis, we expected neuronal responses with negative feedback to build up with repeated false allegations of erroneous performance . We therefore included a separate parametric modulator to the two conditions coding negative feedback. Also see figure 2 in the main text.

The figure also displays parametric modulators coding linear effects of time (e.g. due to fatigue). The linear effects of time were not used in group level analyses but served to explain additional variance in the first level data. Both parametric modulators were convolved with the hemodynamic response function as they were used to explain neuronal processing through the BOLD effect.
