# Supplementary material for: Irritability in pre-clinical Huntington's disease
Source: Neuropsychologia. 2010 Jan;48(2):549–57. doi: 10.1016/j.neuropsychologia.2009.10.016 (PMC2809920; doi:10.1016/j.neuropsychologia.2009.10.016)
Supplement: Supplementary file 2 [file mmc2.doc]

# Supplement 2: Voxel based analysis of T1-weighted data

All T1 weighted images were analysed using SPM5 ([www.fil.ion.ucl.ac.uk/spm/](http://www.fil.ion.ucl.ac.uk/spm/)). Images were segmented into grey matter, white matter and normalised to MNI space using a unified approach (Ashburner and Friston, 2005).This technique employs prior tissue probability maps for each tissue class that code the probability of each voxel belonging to a given tissue class. The intensity distribution of voxels from each class is modelled as a mixture of Gaussians. After an initial affine normalisation step the tissue probability maps are then warped to fit individual T1 images. Parameters for bias correction, tissue classification and spatial normalisation are iteratively estimated from the same generative model. An additional step, usually referred to as modulation, is included to compensate for the effect of spatial normalisation. This step involves multiplying the spatially normalised segmented images by their relative volume before and after spatial normalisation (Ashburner and Friston, 2000). After this step, the values of each voxel represent a measure of the local volume of that tissue class. Finally, we smoothed the data using an isotropic Gaussian smoothing kernel of 12 mm (full width at half maximum). This was done to render the data more normally distributed and to account for the inexact nature of the normalisation process. Data between the two groups was compared with two sample t-tests. We display the results (supplementary Fig 2) at an exploratory threshold of p=0.01 (uncorrected).


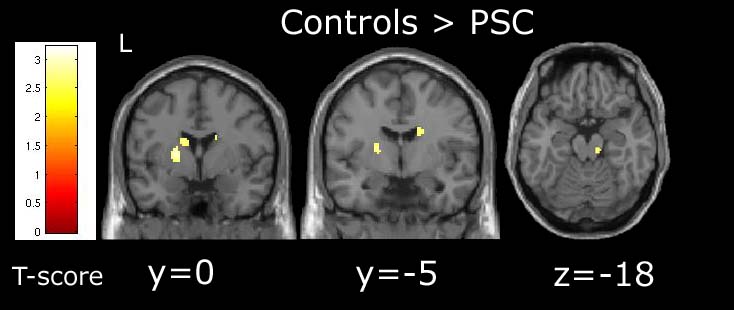


Supplementary Fig. 2. Results when testing for areas with a greater local grey matter volume in controls compared to PSC. Results are overlaid on a single subject’s image in MNI-space. The middle and right panels show sections corresponding to Figures 2 und 3 of the main manuscript.

The striatum bilaterally shows decreased local grey matter volume in PSCs compared to controls. Stronger effects in the left hemisphere are in line with previous work (Klöppel et al., 2008; Mühlau et al., 2007). Note that none of the areas where significant after correcting for multiple comparisons either within one of the regions of interest (OFC or amygdala) or across the whole brain.

References:

Ashburner J, Friston KJ (2000). Voxel-based morphometry--the methods. Neuroimage, 11, 805-21.

Ashburner J, Friston KJ (2005). Unified segmentation. Neuroimage, 26, 839-51.

Klöppel S, Draganski B, Golding CV, Chu C, Nagy Z, Cook PA, Hicks SL, Kennard C, Alexander DC, Parker GJ, Tabrizi SJ, Frackowiak RS (2008). White matter connections reflect changes in voluntary-guided saccades in pre-symptomatic Huntington's disease. Brain, 131, 196-204.

Mühlau M, Gaser C, Wohlschlager AM, Weindl A, Stadtler M, Valet M, Zimmer C, Kassubek J, Peinemann A (2007). Striatal gray matter loss in Huntington's disease is leftward biased. Movement Disorders, 22, 1169-73.
