# Supplementary material for: Irritability in pre-clinical Huntington's disease
Source: Neuropsychologia. 2010 Jan;48(2):549–57. doi: 10.1016/j.neuropsychologia.2009.10.016 (PMC2809920; doi:10.1016/j.neuropsychologia.2009.10.016)
Supplement: Supplementary file 3 [file mmc3.doc]

Supplementary material 3: Correlation matrix of questionnaires using in the study based on Pearson’s correlation coefficient. Two tailed tests are reported throughout to improve clarity.

**Correlations**

|  | | NART | BIS-11 | Snaith, total | Snaith, irritability subscore | STAI (state) | STAI (trait) | BDI |
| --- | --- | --- | --- | --- | --- | --- | --- | --- |
| NART | Pearson Correlation | 1 | -,477(**) | ,076 | ,028 | ,054 | ,085 | ,116 |
|  | Sig. (2-tailed) |  | ,007 | ,686 | ,882 | ,774 | ,647 | ,533 |
|  | N | 31 | 31 | 31 | 31 | 31 | 31 | 31 |
| BIS-11 | Pearson Correlation | -,477(**) | 1 | ,423(*) | ,377(*) | ,521(**) | ,585(**) | ,515(**) |
|  | Sig. (2-tailed) | ,007 |  | ,018 | ,037 | ,003 | ,001 | ,003 |
|  | N | 31 | 31 | 31 | 31 | 31 | 31 | 31 |
| Snaith, total | Pearson Correlation | ,076 | ,423(*) | 1 | ,937(**) | ,786(**) | ,680(**) | ,764(**) |
|  | Sig. (2-tailed) | ,686 | ,018 |  | ,000 | ,000 | ,000 | ,000 |
|  | N | 31 | 31 | 31 | 31 | 31 | 31 | 31 |
| Snaith, irritability subscore | Pearson Correlation | ,028 | ,377(*) | ,937(**) | 1 | ,713(**) | ,593(**) | ,699(**) |
|  | Sig. (2-tailed) | ,882 | ,037 | ,000 |  | ,000 | ,000 | ,000 |
|  | N | 31 | 31 | 31 | 31 | 31 | 31 | 31 |
| STAI (state) | Pearson Correlation | ,054 | ,521(**) | ,786(**) | ,713(**) | 1 | ,718(**) | ,789(**) |
|  | Sig. (2-tailed) | ,774 | ,003 | ,000 | ,000 |  | ,000 | ,000 |
|  | N | 31 | 31 | 31 | 31 | 31 | 31 | 31 |
| STAI (trait) | Pearson Correlation | ,085 | ,585(**) | ,680(**) | ,593(**) | ,718(**) | 1 | ,735(**) |
|  | Sig. (2-tailed) | ,647 | ,001 | ,000 | ,000 | ,000 |  | ,000 |
|  | N | 31 | 31 | 31 | 31 | 31 | 31 | 31 |
| BDI | Pearson Correlation | ,116 | ,515(**) | ,764(**) | ,699(**) | ,789(**) | ,735(**) | 1 |
|  | Sig. (2-tailed) | ,533 | ,003 | ,000 | ,000 | ,000 | ,000 |  |
|  | N | 31 | 31 | 31 | 31 | 31 | 31 | 31 |

** Correlation is significant at the 0.01 level (2-tailed).

* Correlation is significant at the 0.05 level (2-tailed).
